# Speckle echocardiographic left atrial strain and stiffness index as predictors of maintenance of sinus rhythm after cardioversion for atrial fibrillation: a prospective study

**DOI:** 10.1186/1476-7120-10-48

**Published:** 2012-12-03

**Authors:** Amir Y Shaikh, Abhishek Maan, Umar A Khan, Gerard P Aurigemma, Jeffrey C Hill, Jennifer L Kane, Dennis A Tighe, Eric Mick, David D McManus

**Affiliations:** 1 Department of Internal Medicine, University of Massachusetts Medical School, Worcester, USA; 2 Division of Cardiovascular Medicine, University of Massachusetts Medical School, Worcester, USA; 3 Sanford-Brown College of Boston, Boston, USA; 4 Department of Quantitative Health Sciences, University of Massachusetts Medical School, Worcester, USA; 5 Department of Medicine, Cardiology Division, Electrophysiology Section, University of Massachusetts Medical Center, 55 Lake Avenue North, Worcester, MA, 01655, USA

**Keywords:** Arrhythmia, Echocardiography, Strain, Stiffness

## Abstract

**Background:**

Echocardiographic left atrial (LA) strain parameters have been associated with atrial fibrillation (AF) in prior studies. Our goal was to determine if strain measures [peak systolic longitudinal strain (LAS) and stiffness index (LASt)] changed after cardioversion (CV); and their relation to AF recurrence.

**Methods and results:**

46 participants with persistent AF and 41 age-matched participants with no AF were recruited. LAS and LASt were measured before and immediately after CV using 2D speckle tracking imaging (2DSI). Maintenance of sinus rhythm was assessed over a 6-month follow up. Mean LAS was lower, and mean LASt higher, in participants with AF before CV as compared to control group (11.9 ± 1.0 vs 35.7 ± 1.7, p<0.01 and 1.31 ± 0.17 vs 0.23 ± 0.01, p<0.01, respectively). There was an increase in the mean LAS immediately after CV (11.9 ± 1.0 vs 15.9 ± 1.3, p<0.01), whereas mean LASt did not change significantly after CV (p=0.62). Although neither LAS nor LASt were independently associated with AF recurrence during the follow-up period, change in LAS after cardioversion (post-CV LAS – pre-CV LAS) was significantly higher among individuals who remained in sinus rhythm when compared to individuals with recurrent AF (3.6 ± 1.1 vs 0.4 ± 0.8, p=0.02).

**Conclusions:**

LAS and LASt differed between participants with and without AF, irrespective of the rhythm at the time of echocardiographic assessment. Baseline LAS and LASt were not associated with AF recurrence. However, change in LAS after CV may be a useful predictor of recurrent arrhythmia.

## Introduction

Atrial fibrillation (AF) is the most common arrhythmia encountered, and the population burden of AF is increasing [1-7]. Pathological atrial structural remodeling characterized, by fibrosis and myofiber disarray, plays a critical role in AF initiation and maintenance [1,8-10].

Increased left atrial (LA) size on echocardiogram is associated with incident AF, recurrent AF, stroke and mortality [4,11-15]. Nevertheless, LA size only slightly improves AF risk prediction [16]. More recently, atrial myocardial deformation properties measured as strain, strain rate and stiffness (E/E’/strain) by tissue Doppler Imaging (TDI) and 2D speckle tracking imaging (2DSI) have shown promise as markers of atrial structural remodeling and as predictors of AF [1-5,17-19]. 2DSI measures local atrial myocardial deformation and unlike TDI, is angle independent, and unaffected by cardiac translation or tethering effect [4,15].

Preliminary work suggests that LA strain parameters are independent predictors of recurrence of AF in subjects undergoing cardioversion (CV) or catheter ablation [6,9,20-22]. The purpose of this prospective investigation was to determine if LA stiffness index (LASt), and/or peak systolic longitudinal LA strain (LAS) changed after CV in subjects with persistent AF; and their relation as predictors of maintenance of sinus rhythm. To our knowledge, this is the first study evaluating the role of LASt in AF recurrence after CV.

## Methods

### Patient population

This study was approved by the University of Massachusetts Medical School Institutional Review Board. Between June of 2010 and May of 2011, 46 participants with persistent AF (duration of less than 12 months) undergoing a clinically indicated elective CV for AF and 41 age-matched controls were recruited. Written-informed consent was obtained prior to enrollment. Exclusion criteria were: history of prior cardiac surgery, history of atrial flutter, history of paced atrial or ventricular rhythm, history of aortic or mitral valvular prosthesis, history of significant (moderate or greater) mitral insufficiency or stenosis, history of atrial or ventricular thrombus, history of atrial septal defect, pregnancy, age less than 18, inability to give consent and failure of CV. Five participants were excluded from the study since they failed cardioversion. Control subjects were subjects who had undergone an echocardiogram for varied indications but had no history of AF. A standard 2-Dimensional (2D) transthoracic echocardiography (TTE) was performed prior to elective CV and was repeated within 24 h post-CV in sinus rhythm. CV was performed as per ACC/AHA/ESC guidelines with direct-current, synchronized shock in fasting subjects after intravenous sedation [23].

### Echocardiography

A standard TTE was performed using a Vivid 7 (GE Healthcare, Waukesha, WI) and a 1.7/3.4 MHz tissue harmonic transducer with a frame rate of at least 50 frames per second. Machine settings were manually adjusted to optimize 2D endocardial and myocardial gray scale definition. Care was taken to obtain true apical images using standard anatomic landmarks in each view and not foreshorten the left ventricle or left atrium. All images were acquired at end-expiratory apnea. Loops of 5 cardiac cycles were stored digitally and analyzed offline using a customized software package (EchoPAC, GE Healthcare).

#### Two-dimensional and Doppler echocardiographic variables

LA diameter (LAD) was measured at end-systole along the parasternal long-axis view. Maximum LA volume (end-ventricular systole) was calculated from apical 4- and apical 2-chamber views of the LA using the biplane method of disks, as is recommended by the American Society of Echocardiography [24]. Left ventricular end-systolic volume, left ventricular end-diastolic volume, and left ventricular ejection fraction (LVEF) were measured. Mitral flow velocity (E) was assessed by pulsed-wave Doppler from the apical 4 chamber view by placing a sample volume between the tips of the mitral leaflets in diastole and recording at a sweep speed of 100 mm/s. Peak flow velocities were measured and averaged from five consecutive cycles. The average of lateral and medial mitral annulus early diastolic velocity (E’) was measured using TDI. The E/E’ ratio was used to estimate filling pressures [25].

#### Speckle tracking imaging

**Figure 1:**
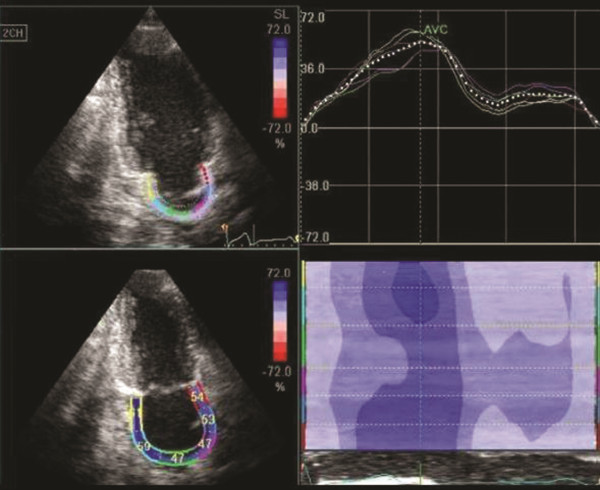
Assessment of peak systolic longitudinal left atrial strain (LAS) by 2D speckle tracking imaging (2DSI).

Offline 2DSI technique was used to calculate LAS in individual LA regions. For 2DSI, a line was manually drawn along the LA endocardium at peak systole corresponding with the aortic valve closure. The software then automatically generated additional lines near the atrial epicardium, with a region of interest default width that was manually adjusted. Before processing, a cine loop preview feature was utilized to visually confirm that the internal line follows the LA endocardium throughout the cardiac cycle. If tracking of the LA endocardium was found to be unsatisfactory, then manual adjustments or changing software parameters (e.g., region of interest size or smoothing functions) were made. For analysis of the LAS, region of interest was divided into 6 segments of the LA wall in each 4 and 2 chamber views and were manually tracked frame by frame to maintain the position within the LA wall. LAS was measured at each segment divided from the annulus to the roof of LA (Figure 1). The LAS of the 6 segments was averaged for analysis. Approximately 90.5% of the individual segments throughout the study had adequate speckle tracking, and were included for strain analysis. The other 9.5% of the individual segments had inadequate speckle tracking due to either poor image quality or pulmonary vein openings, and were excluded from the analysis. Inter-observer variability expressed as a coefficient of variation, was assessed by analyzing 10 randomly chosen subjects by 2 independent investigators. For intra-observer variability, 10 randomly chosen participants were analyzed by the same investigator twice with a minimum gap of a month. The intra-observer and inter-observer variability for LAS were 4% and 7% respectively, and for LASt were 5% and 7% respectively.

In a recent study the validity and accuracy of LASt was examined using both invasive LA pressure, and non-invasive surrogates of left atrial pressure (E/A, E/E’, and E/V_p_) [21]. The calculation of LASt using E/E’ had closest correlation to LASt derived by invasive LA pressures [21]. In our study we calculated the LASt as (E/E’/LAS).

### Atrial fibrillation

AF at baseline was diagnosed on a 12-lead electrocardiogram. All participants were discharged with a 30-day continuous electrocardiography recording monitor (Heartrak ECAT(External Cardiac Ambulatory Telemetry); AMI Cardiac Monitoring; Sandy Spring, Maryland) after CV. Any abnormal electrical activity recordings were transmitted to the electrophysiologist on a daily basis even if not triggered by subjects and asymptomatic. All AF recurrences were adjudicated by an experienced cardiac electrophysiologist (DDM). Using an electronic medical record chart review, subjects were followed for 6 months for interim hospitalizations, emergency room visits and outpatient visits. Any evidence of AF based on electrocardiographic findings was considered as recurrence.

### Statistical analysis

The data were initially analyzed for normality. Chi square tests for categorical variables and Student’s *t* test for continuous variables were used to examine potential differences between study groups. Wilcoxon rank sum test for paired variables and Mann–Whitney *U* test for unpaired variables were used for data that did not have a normal distribution. Ordinal variables were tabulated as mean ± 2 SEM or with the confidence intervals of 5^th^ and 95^th^ percentiles.

Area under the Receiver Operating Curve (ROC), univariate and multivariate analysis were performed to describe the prognostic value of strain parameters for prediction of sinus rhythm maintenance after CV. Cutoff for p values in univariate analysis was 0.3 to enter multivariate analysis; and was considered statistically significant in multivariate analysis only if p was < 0.05. Sensitivity, specificity and optimal cut-off values were determined from the ROC curve data. All the analyses were performed with commercially available packages for (SPSS, Version 20.0, SPSS Inc., Chicago, IL, USA; and GraphPad Prism version 5.00 for Windows, GraphPad Software, San Diego, California, USA).

## Results

### Clinical characteristics of AF and controls

The general clinical characteristics are recorded in Table 1. Subjects in AF group were predominantly male, and had a higher incidence of obesity, hypertension and heart failure as compared to the control group.

**Table 1:** Baseline clinical characteristics of patients with atrial fibrillation (AF) as compared to the control group

	**AF (41) n (%)**	**Controls (41) n (%)**	**P value**
Mean age (years)	64.5 ± 4.0	61.7 ± 2.4	0.12
Sex (M/F)	29/12	13/28	<0.01
BMI (Kg/m^2^)	32.5 ± 2.3	25.8 ± 1.6	<0.01
**Comorbid conditions:**			
HTN	30 (73.2)	16 (39)	<0.01
CAD	14 (34.1)	4 (9.7)	<0.01
Systolic HF	13 (31.7)	0	<0.01
CVA	1 (2.4)	0	1.0
DM	11 (26.8)	4 (9.7)	0.08
Dyslipidemia	24 (58.5)	9 (21.9)	<0.01
**Medications:**			
Statins	22 (53.6)	6 (14.6)	<0.01
Beta blockers	27 (65.8)	9 (21.9)	<0.01
ACE-i/ARB	24 (58.5)	8 (19.5)	<0.01
CCB	17 (41.5)	5 (12.2)	<0.01
Digoxin	4 (9.7)	0	0.11
Diuretics	21 (51.2)	5 (12.2)	<0.01
ASA	30 (73.2)	5 (12.2)	<0.01
Clopidogrel	2 (4.8)	0	0.49
Warfarin	28 (68.3)	3 (7.3)	<0.01
Heparin	4 (9.7)	0	0.11
Amiodarone	8 (19.5)	0	<0.01
Sotalol	5 (12.2)	0	0.05
Other anti-arrhythmics	4 (9.7)	0	0.11

BMI – Body Mass Index, HTN – Hypertension, CAD – Coronary artery disease, HF- Heart failure, DM – Diabetes Mellitus, CVA – Cerebrovascular accident, ACE-i/ARB – Angiotensinogen converting enzyme inhibitors/Angiotensin receptor blockers, CCB – Calcium channel blockers, ASA - Aspirin.

### Comparison of echocardiographic and strain parameters in AF and controls pre and post CV

**Figure 2:**
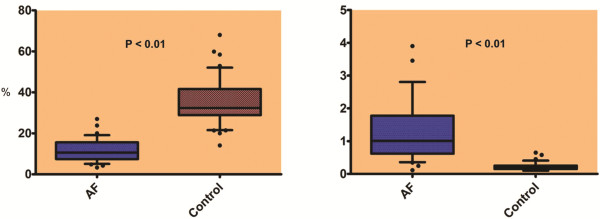
Comparison of mean LAS (left) and of left atrial stiffness index (LASt) (right) at baseline between the AF group and control group.

**Figure 3:**
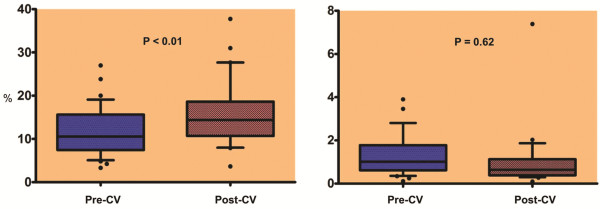
Comparison of mean LAS (left panel) and of LASt (right panel) in subjects with AF pre and post cardioversion (CV).

The echocardiographic variables and strain parameters in participants with AF and controls with no history of AF are displayed in Table 2. Mean LAS was significantly lower in subjects with AF, pre-CV as compared to the control group (11.9 ± 1.0 vs 35.7 ± 1.7, p<0.01) (Figure 2). Mean LAS was lower (15.9 ± 1.3 vs 35.7 ± 1.7, p <0.01), and mean LASt higher (1.1 ± 0.3 vs 0.2 ± 0.01, p<0.01), in participants with AF after CV as compared to participants with no history of AF (Figure 2). Among participants with AF, there was a significant increase in mean LAS (11.9 ± 1.0 vs 15.9 ± 1.3, p<0.01), but no change in LASt (p=0.62), after CV (Figure 3).

**Table 2:** Echocardiographic and strain variables of patients with atrial fibrillation (AF) as compared to the control group

**Echo variables**	**AF (n=41)**			**Controls (n=41)**	**P value**
LVIDd, mm	50.2 ± 2.6			44.8 ± 1.8	<0.01
LVIDs, mm	35.6 ± 3.2			28.0 ± 1.8	<0.01
STd, mm	10 ± 0.6			8.9 ± 0.6	<0.01
PWTd, mm	10 ± 0.6			8.1 ± 0.6	<0.01
LVEF (%)	54.0 ± 3.6			65.1 ± 1.0	<0.01
LAD, mm		45.9 ± 4.0			33.4 ± 2.0	<0.01
LAVI, ml/M^2^	34.7 ± 4.0			23.4 ± 3.2	<0.01	
E/E’	10.7 ± 1.8			7.6 ± 0.8	<0.01	
	**Pre- CV**	**Post- CV**	**P value**			
2CLAS, %	12.7 (10.2 – 15.2)	16.5 (13.2 – 19.9)	0.01	38.3 (33.1 – 43.5)	<0.01	
4CLAS, %	11.0 (8.6 – 13.5)	15.2 (12.4 – 18.0)	<0.01	33.1 (29.6 – 36.6)	<0.01	
LAS, %	11.9 (9.8 – 13.9)	15.9 (13.1 – 18.7)	<0.01	35.7 (32.1 – 39.3)	<0.01	
2CLASt	1.06 (0.81 – 1.32)	1.25 (0.16 – 2.33)	0.67	0.24 (0.19 – 0.28)	<0.01	
4CLASt	1.57 (1.04 – 2.10)	0.85 (0.61 – 1.09)	0.02	0.24 (0.20 – 0.28)	<0.01	
LASt	1.32 (0.97 – 1.67)	1.05 (0.44 – 1.66)	0.62	0.23 (0.19 – 0.27)	<0.01	

LVIDd – Left ventricular internal diameter at end- diastole, LVIDs – Left ventricular internal diameter at end- systole, STd – Interventricular septum thickness at end-diastole, PWTd – Left ventricular posterior wall thickness at end-diastole, LVEF – Left ventricular ejection fraction, LAD – Left atrial diameter, LAVI – Left atrial volume index, E = early filling velocity of transmitral Doppler flow, E’ = Average early diastolic velocity of medial and lateral mitral annulus, 2CLAS - 2 chamber average peak systolic strain, 4CLAS - 4 chamber average peak systolic strain, LAS – Mean peak systolic strain of 2C and 4C, 2CLASt - 2 chamber left atrial stiffness, 4CLASt – 4 chamber left atrial stiffness, LASt – Mean left atrial stiffness of 2C and 4C.

### Clinical characteristics of the maintained sinus rhythm (MSR) and AF recurrence (AFR) groups

Twenty-seven (66%) of participants undergoing CV for persistent AF remained in normal sinus rhythm throughout the 6-month follow-up period, whereas 14 (34%) experienced at least 1 recurrence of AF. There was no significant difference in age, sex, body mass index (BMI), comorbidities or medications, especially anti-arrhythmic drugs between both the groups (Table 3). Three out of fourteen subjects had AF recurrence within 24 h. Eight out of fourteen subjects had AF recurrence within 30 days. The remaining three subjects had AF recurrence between one and six months. Amongst the subjects who had a recurrence within 30 days, the mean AF burden was 46.13%. None of the study subjects had new onset stroke or transient ischemic attack during the 6 month follow up.

**Table 3:** Baseline clinical characteristics of patients in the maintained sinus rhythm (MSR) group as compared to the atrial fibrillation recurrence (AFR) group

	**MSR (27) n (%)**	**AFR (14) n (%)**	**P value**
Mean age (years)	65 ± 5.0	64 ± 6.8	0.9
Sex (M/F)	21/6	8/6	0.28
BMI (Kg/m^2^)	32 ± 3.0	34 ± 3.6	0.26
**Comorbid conditions:**			
HTN	21 (77.8)	12 (85.7)	0.69
CAD	9 (33.3)	5 (35.7)	1.0
Systolic HF	11 (40.7)	3 (21.4)	0.3
CVA	0 (0)	1 (7.1)	0.34
DM	5 (18.5)	6 (42.8)	0.14
Dyslipidemia	16 (59.2)	8 (57.1)	1.0
**Medications:**			
Statins	14 (51.8)	8 (57.1)	1.0
Beta blockers	20 (74.1)	7 (50.0)	0.17
ACE-i/ARB	14 (51.8)	11 (78.5)	0.17
CCB	12 (44.5)	6 (42.8)	1.0
Digoxin	2 (7.4)	2 (14.3)	0.59
Diuretics	16 (59.2)	5 (35.7)	0.19
ASA	18 (66.7)	12 (85.7)	0.28
Clopidogrel	1 (3.7)	1 (7.1)	1.0
Warfarin	18 (66.7)	10 (71.4)	1.0
Heparin	1 (3.7)	3 (21.4)	0.11
Amiodarone	7 (25.9)	1 (7.1)	0.23
Sotalol	2 (7.4)	3 (21.4)	0.32
Other anti-arrhythmics	3 (11.1)	1 (7.1)	1.0

BMI – Body Mass Index, HTN – Hypertension, CAD – Coronary artery disease, HF- Heart failure, DM – Diabetes Mellitus, CVA – Cerebrovascular accident, ACE-i/ARB – Angiotensinogen converting enzyme inhibitors/Angiotensin receptor blockers, CCB – Calcium channel blockers, ASA - Aspirin.

### Comparison of echocardiographic and strain parameters in the MSR and AFR group

**Figure 4:**
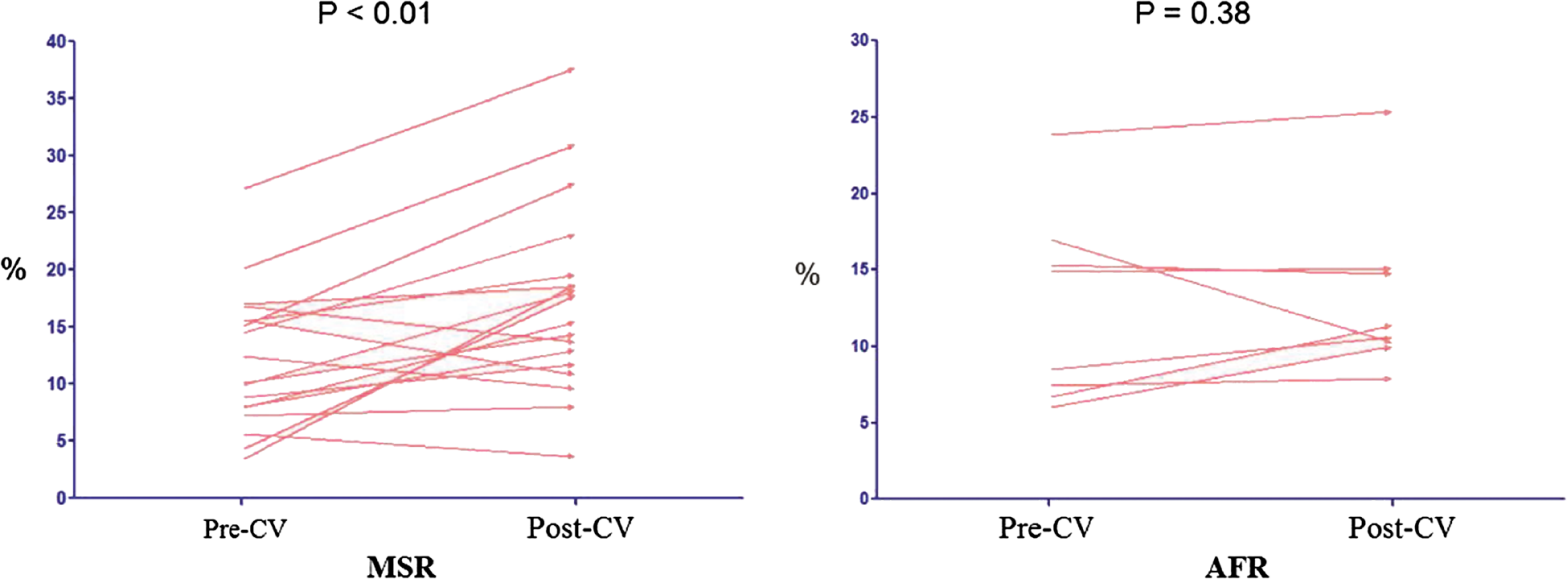
Post-CV mean LAS as compared to pre-CV mean LAS in the maintained sinus rhythm group (MSR) versus the atrial fibrillation recurrence group (AFR).

The echocardiographic variables and strain parameters of MSR and AFR groups are recorded in Table 4. There was no significant difference in mean pre-CV LAS between both the groups (11.8 ± 1.3 vs 12.0 ± 1.6, p=0.95). Mean post-CV LAS was higher in the MSR group as compared to the AFR group (17.0 ± 1.8 vs 13.5 ± 1.7, p=0.19). In the MSR group, mean LAS after CV was higher as compared to the pre-CV LAS (11.8 ± 1.3 vs 17.0 ± 1.8, p<0.01). In contradistinction, participants who had a recurrence of AF had no significant change in LAS after CV (12.0 ± 1.6 vs 13.5 ± 1.7, p=0.38) (Figure 4). Difference in mean LAS after CV was statistically significant in the MSR group as compared to the AFR group (3.6 ± 1.1 vs 0.4 ± 0.8, p=0.02). There was no significant change in mean LASt after CV in either the MSR or the AFR group (1.28 ± 0.22 vs 1.12 ± 0.41, p=0.92; 1.38 ± 0.26 vs 0.87 ± 0.18; p=0.22) respectively.

**Table 4:** Echocardiographic and strain variables of patients with maintained sinus rhythm (MSR) group as compared to the atrial fibrillation recurrence (AFR) group

**Echo variables**	**MSR (n=27)**			**AFR (n=14)**		**P value**
LVIDd, mm	49.6 ± 2.6			51.5 ± 6.6		0.68
LVIDs, mm	35.8 ± 3.6			35.2 ± 6.6		0.84
STd, mm	10.4 ± 0.8			10.5 ± 0.8		0.61
PWTd, mm	10.1 ± 0.8			10.8 ± 1.0		0.18
LVEF (%)	54.8 ± 4.4			52.5 ± 7.0		0.72
LAD, mm	46.5 ± 3.8			44.7 ± 9.4		0.95
Pre-CV LAVI, ml/M^2^	34.5 ± 5.0			36.5 ± 5.6		0.82
Post-CV LAVI, ml/M^2^	35 ± 5.6			34.2 ± 5.2		0.89
E/E’	9.8 ± 2.2			12.5 ± 2.6		0.07
	**Pre-CV**	**Post-CV**	**P value**	**Pre-CV**	**Post-CV**	**P value**
HR	84.0 (77.6 – 90.6)	66.0 (60.5 – 71.6)	<0.01	87.9 (78.2 – 97.6)	75.7 (69.3 – 82.1)	0.05
LAVI, ml/M^2^	34.5 (29.4 – 39.8)	35 (29.2 – 40.9)	0.87	36.5 (30.9 – 42.1)	34.2 (29.0 – 39.4)	0.19
2CLAS, %	12.8 (9.5 – 16.3)	18.0 (13.4 – 22.8)	0.01	12.4 (8.1 – 16.8)	13.2 (10.7 – 15.8)	0.68
4CLAS, %	10.8 (7.6 – 14.1)	15.9 (12.5 – 19.3)	<0.01	11.6 (7.5 – 15.7)	13.7 (7.7 – 19.9)	0.75
LAS, %	11.8 (9.1 – 14.6)	17.0 (13.2 – 20.8)	<0.01	12.0 (8.3 – 15.7)	13.5 (9.5 – 17.6)	0.38
2CLASt	0.92 (0.63 – 1.20)	1.42 (−0.16 – 2.99)	0.5	1.32 (0.78 – 1.87)	0.83 (0.52 – 1.14)	0.25
4CLASt	1.64 (0.86 – 2.41)	0.83 (0.54 – 1.12)	0.06	1.45 (0.78 – 2.13)	0.92 (0.32 – 1.52)	0.24
LASt	1.28 (0.81 – 1.76)	1.12 (0.25 – 1.99)	0.92	1.39 (0.81 – 1.97)	0.88 (0.43 – 1.33)	0.22

LVIDd – Left ventricular internal diameter at end- diastole, LVIDs – Left ventricular internal diameter at end- systole, STd – Interventricular septum thickness at end-diastole, PWTd – Left ventricular posterior wall thickness at end-diastole, LVEF – Left ventricular ejection fraction, LAD – Left atrial diameter, LAVI – Left atrial volume index, E = early filling velocity of transmitral Doppler flow, E’ = Average early diastolic velocity of medial and lateral mitral annulus, HR – Heart rate, 2CLAS - 2 chamber average peak systolic strain, 4CLAS - 4 chamber average peak systolic strain, LAS – Mean peak systolic strain of 2C and 4C, 2CLASt - 2 chamber left atrial stiffness, 4CLASt – 4 chamber left atrial stiffness, LASt – Mean left atrial stiffness of 2C and 4C.

### LAS and LASt as predictors for maintenance of sinus rhythm

A regression analysis adjusted for age, sex and BMI was performed initially and showed that difference in strain (post-CV LAS – pre-CV LAS) was a predictor of maintenance of sinus rhythm (B coefficient 3.1, p=0.09). Further multivariate regression adjusting for age and BMI, demonstrated that the difference in strain showed a trend towards prediction of MSR (B coefficient 3.7, p = 0.08) (Table 5). ROC curves were plotted for pre-CV LAS (AUC 0.52 ± 0.09), post-CV LAS (AUC 0.38 ± 0.09) and difference in strain (AUC 0.38 ± 0.09). ROC curves for pre-CV LASt had an AUC of 0.43 ± 0.08.

**Table 5:** Univariate and Multivariate predictors of maintenance of sinus rhythm in patients with atrial fibrillation (AF) after cardioversion

**Parameter**	**Univariate regression**	
	**B co-efficient**	**P value**
Age	2.3	0.53
Sex	−0.2	0.14
BMI	−2.4	0.35
Pre-CV LAVI	2.3	0.65
Difference in LAVI	−2.8	0.44
Pre-CV HR	−0.01	0.99
Difference in HR	7.3	0.27
Pre-CV LAS	0.06	0.98
Difference in LAS	3.1	0.09
Pre-CV LASt	−0.2	0.55
Difference in LASt	0.5	0.59
	**Multivariate regression**	
	**B co-efficient**	**P value**
Sex	−0.2	0.32
Difference in HR	4.7	0.49
Difference in LAS	3.7	0.08

## Discussion

In this prospective clinical study involving 82 participants, we found that mean LAS was lower and mean LASt higher in subjects with AF as compared to those in normal sinus rhythm and no history of AF. We also observed that, although mean LAS improved immediately after CV in subjects with AF, it failed to normalize completely and remained lower in subjects with a history of AF than the control group. Mean LASt did not significantly change after CV. Compared with individuals who experienced a recurrence of AF over the follow-up period, those who remained in normal sinus rhythm were more likely to experience a significant improvement in the LAS after CV. Difference in strain (post-CV LAS – pre-CV LAS) showed a trend towards prediction of maintenance of sinus rhythm after cardioversion.

### Atrial myocardial deformation properties and AF

Normal LA function comprises three components: reservoir function, serving to store blood from the pulmonary veins; conduit function, wherein blood passes through the left atrium from the pulmonary veins to the left ventricle in early diastole; and booster function, where atrial contraction serves to increase end diastolic ventricular fiber stretch [2]. Several studies have demonstrated that atrial structural remodeling during AF is due to a variable degree of fibrosis, atrial myocyte hypertrophy, myofiber disarray and apoptosis [8,10]. Atrial strain parameters have been proven to correlate significantly with the underlying fibrosis and have been validated against sonomicrometry and tagged MRI, thus providing a comprehensive real time quantitative assessment of regional atrial myocardial deformation [15,26]. However, strain and strain rate can be influenced by loading conditions and may be influenced by rhythm irregularity, irrespective of degree of underlying fibrosis [3,9].

Preliminary work suggests that LA strain parameters are independent predictors of recurrence of AF in subjects undergoing cardioversion (CV) or catheter ablation [6,9,20-22]. Accordingly, the study was undertaken to better understand to what extent systolic strain is influenced by the atrial rhythm. We also evaluated the role of these strain variables in predicting the recurrence of AF.

### Recovery of left atrial function after CV and re-established sinus rhythm

Our finding that LAS was significantly lower in subjects with AF as compared to controls was similar to the findings of prior studies [3,4,6,22]. As has previously been reported in the published literature, LAS increased significantly immediately after CV. However, mean LAS did not normalize completely [6,7,27,28]. This is consistent with the findings of a 3-year follow-up study, which demonstrated that although the LA contractility improved after successful CV, it remained modestly impaired [27]. In contrast to this work, however, we measured LAS immediately after CV instead of 1 month after CV. The improvement in LA strain after CV or catheter ablation has been previously attributed to ‘reverse atrial remodeling [27]. The immediate improvement in LAS after cardioversion in individuals with AF enrolled in our study makes structural remodeling less likely and restoration of atrial contraction as the more likely cause of the change in LAS. The fact that LAS measured immediately after CV did not improve to levels seen among individuals with no history of AF could have resulted from “atrial stunning” or from persistent mechanical and/or LA structural abnormalities.

As has been previously reported [21], baseline LASt was significantly higher among participants with AF as compared to participants with no AF, likely secondary to decreased LA compliance in subjects with AF. Interestingly, there was no change in LASt immediately after CV. In comparison with LAS, the fact that LASt values were similar in AF and after cardioversion in sinus rhythm, suggests that LASt is a more reliable index of the underlying structural characteristics of the LA than LAS. Stiffness being directly proportional to LA size, it is not surprising that LASt did not change in the short term, as there was no opportunity for reverse remodeling of the LA.

### Prediction of maintenance of sinus rhythm

The degree of impairment in atrial compliance, as assessed by LAS, has been reported to relate to maintenance of sinus rhythm after CV or catheter ablation in subjects with persistent AF [2,3,6,9]. However, our study findings showed no significant difference for the pre- and post-CV LAS and LASt values between the MSR and AFR group. Also unlike prior studies, we did not find baseline LAS or LASt to be an independent predictor of maintenance of sinus rhythm [21]. We found that the change in LAS was associated with maintenance of sinus rhythm. Although our finding did not meet statistical significance, this finding was similar to Schneider et al. who also demonstrated that LAS increased in subjects with MSR during a three month follow up in contrast to subjects with AFR [6]. Due to the low rates of post-conversion recurrent AF, we acknowledge that further studies are needed in order to have the appropriate statistical power to address this issue.

The results of our study could be influenced by several factors. Even though we used a continuous event monitor for one month, we could have missed asymptomatic AF recurrences between month two to six of follow-up. The duration of persistent AF may have played a role in altering the predictive probability of strain for AF recurrence. In contrast to our study, which only included participants with persistent AF, most of the studies that have shown that lower LAS is associated with higher recurrence rates have recruited subjects with both paroxysmal and persistent AF. Since Schneider et al. have reported lower strain values in subjects with persistent AF as compared to paroxysmal AF, it is possible that our exclusion of participants with paroxysmal AF may have influenced our findings [6]. Also, the platform utilized to analyze strain variables can possibly influence the magnitude of the strain values thus leading to differing results. Evaluation of LASt and LAS in a larger sample of participants with both paroxysmal and persistent AF is warranted to more fully evaluate the potential associations between LA strain measures and AF recurrences.

### Study limitations

A long-term prospective study with repeated strain measurement over time would be ideal to assess the effects of reverse atrial modeling on strain and stiffness. Studies with larger sample size with subgroup analysis of paroxysmal and persistent AF would be beneficial in further evaluating role of strain as predictive markers for AF recurrence.

### Clinical implication

Recent studies suggest LA strain as a predictor of stroke risk and cardiovascular outcomes in patients with AF [29,30]. A better understanding of atrial physiology might potentially permit targeted strategies to prevent AF recurrence, thereby decreasing the risk of major adverse cardiovascular outcomes. While baseline atrial strain does not appear to be a predictor of the maintenance of sinus rhythm, our data do suggest the hypothesis that improvement in strain following CV is a favorable prognostic sign.

## Conclusion

We observed that LAS and LASt differed between participants with and without AF, irrespective of the rhythm at the time of echocardiographic assessment; to our knowledge, ours is the first study analyzing left atrial stiffness index before and after cardioversion. Unlike prior studies, we did not find LAS or LASt at baseline to be an independent predictor of AF recurrence. However, our results suggest that change in LAS after CV may be a useful predictor of recurrent arrhythmia. Further long term follow up studies are required to establish the role of this stiffness index utilizing non-invasive surrogate markers of left atrial filling pressure.

## Abbreviations

AF: Atrial fibrillation; LA: Left atrial; TDI: Tissue doppler imaging; 2DSI: 2D Speckle tracking imaging; CV: Cardioversion; LAS: Left atrial peak systolic longitudinal strain; LASt: Left atrial stiffness index; TTE: Trans-thoracic echocardiography; LAD: Left atrial diameter; LVEF: Left ventricular ejection fraction; ROC: Receiver operating curve; BMI: Body mass index; MSR: Maintenance of sinus rhythm; AFR: Atrial fibrillation recurrence; AUC: Area under the curve.

## Competing interests

The author declares that he has no competing interests

## Authors’ contributions

AYS - Concept/design, Data analysis/interpretation, Drafting article, Critical revision of article, Approval of article, Statistics, Data collection, Other. AM - Drafting article, Critical revision of article, Approval of article, Data collection. UAK - Critical revision of article, Approval of article, Data collection. GPA - Concept/design, Data analysis/interpretation, Drafting article, Critical revision of article, Approval of article, Statistics, Data collection, Other. JCH - Critical revision of article, Approval of article, Statistics, Data collection, Other. JLK - Approval of article, Data collection, Other. DAT - Critical revision of article, Approval of article, Statistics, Other. EM - Critical revision of article, Approval of article, Statistics. DDM - Concept/design, Data analysis/interpretation, Drafting article, Critical revision of article, Approval of article, Statistics, Funding secured by, Data collection, Other. All authors read and approved the final manuscript.

## Funding

This study was funded by the Cardiology Division, University of Massachusetts Medical School, by the National Heart, Lung and Blood Institute (1U01HL105268-01) and by KL2RR031981.

## Financial disclosures

AYS – None; AM – None; UAK – None; GPA – None; JCH – consulting contract with Philips Healthcare; JLK – None; DAT – None; EM – None; DDM – Research funding support: Biotronik, Inc. and Philips Healthcare, Inc. All authors contributed equally to this manuscript.
